# Supplementary material for: High-throughput deep learning variant effect prediction with Sequence UNET
Source: Genome Biol. 2023 May 9;24:110. doi: 10.1186/s13059-023-02948-3 (PMC10169183; doi:10.1186/s13059-023-02948-3)
Supplement: Supplementary file 1 — Additional file 1: Supplementary Information. This file contains a longer description of the python packages, supplementary analyses, and all the supplementary figures and relevant discussion. It includes details of the performance of the base PSSM prediction and frequency classifier model and discussions of dataset biases and the effect of variable padding. It also contains some more description of the python packages. [file 13059_2023_2948_MOESM1_ESM.docx]

High-throughput deep learning variant effect prediction with Sequence UNET: Supplementary Information

Alistair S. Dunham^1,2^, Pedro Beltrao^1,3^, Mohammed AlQuraishi^4^

1 - European Molecular Biology Laboratory, European Bioinformatics Institute (EMBL-EBI), Wellcome Genome Campus, Hinxton, Cambridgeshire CB10 1SD, UK.

2 - Wellcome Sanger Institute, Wellcome Genome Campus, Hinxton, Saffron Walden CB10 1RQ

3 - Department of Biology, Institute of Molecular Systems Biology, ETH Zurich, Zurich 8093, Switzerland

4 - Department of Systems Biology, Columbia University, New York, NY, USA

# Python Packages

We developed the Sequence UNET python package [1,2] to help users easily apply the model to other problems. This provides a python interface to download pretrained models from BioStudies [3], train and extend new versions of the model and make predictions on new datasets. It supports sequences in string and BioPython sequence format, meaning most common formats can easily be imported, and sequence/structural data from ProteinNet, which contains most PDB entries and can be filtered and manipulated with ProteinNetPy. It also includes scripts to run the model on new proteins without coding, supporting fasta and ProteinNet input formats. We also developed the ProteinNetPy package [4,5] to parse, process and manipulate ProteinNet data.

# Position specific variant frequency prediction

We first trained Sequence UNET to predict the frequency of each possible mutation in a sequence, and therefore its PSSM. This matrix compares the prevalence of each amino acid at each position to background amino acid frequencies, giving a measure of how well each substitution would be tolerated. The Kullbeck-Liebler divergence between the vector of predicted and true frequencies of each position was used as the loss function and a softmax activation function was used in the final layer to ensure the variant frequencies at each position sum to 1. The results from hyperparameter optimisation for this mode are shown in **Fig. S1**. The PSSMs predicted by this model are very similar to those derived from multiple sequence alignments (**Fig. S2A-B**). The average difference between predicted and true values in the ProteinNet CASP12 test set was 0.04, although this is quite variable ($\sigma=0.072$). Interestingly, it is often the wild-type amino acid that is most incorrectly predicted (WT: $\mu=0.18$, $\sigma=0.184$, Missense: $\mu=0.033$, $\sigma=0.05$), which suggests the model is not able to learn how common the wild-type is as well as it learns what variants are tolerated or rejected.

SPBuild [6] is a recurrent neural network model that makes PSSM predictions and was shown to outperform similar de novo profile generation methods such as CSBuild [7,8] and RPS-Blast [9], so provides a good comparison for state-of-the-art PSSM prediction from sequence. It is also a good general comparison between Sequence UNETs architecture and recurrent models, which were previously the standard approach to sequence-based problems. We also compare to ESM-1b [10,11], which provides a good comparison to large transformer based general protein language models. These are trained by learning to predict which amino acid occurs at each position, meaning they produce a vector of propensities for each amino acid at each position that relates to the PSSM. However, language models are generally used for the position representations they produce, which are used as a general base for task specific models. To compare to this usage, we also trained a simple softmax layer to predict PSSM frequencies from ESM-1b representations, based on the ProteinNet CASP12 95% thinning data.

The correlation between predictions and true values in the ProteinNet test set (**Fig. S2C**) suggests Sequence UNET ($\rho=0.451$) outperforms SPBuild ($\rho=0.332$), baseline evolutionary expectations ($\rho=0.333$) and ESM-1b (ESM1b top model $\rho=0.237$, ESM1b logits $\rho=0.057$), with structural features increasing correlation slightly ($\rho=0.472$). Interestingly base ESM-1b logits strongly correlate with variant frequencies ($\rho=0.551$) but not the PSSM scores, which are normalised against overall amino acid frequencies. This may be because it is trained to identify the most likely amino acid and cannot differentiate magnitudes of rare variants, which are more important for PSSM scores. Sequence UNET and the ESM-1b top model are consistently most likely to be within 1 unit of the true value, be closest to the true value or both (**Fig. S2D**). ESM-1b performs better on these metrics than Pearson correlation, perhaps because it often correctly predicts the frequent low magnitude PSSM scores but fails to predict the high impact variants, which have more impact on correlation than these metrics. These results demonstrate that Sequence UNET improves on the current state of the art for specific de novo PSSM profile prediction and show that the model architecture can outperform LSTM recurrent models while being much more computationally efficient. They also suggest the model compares favourably to much more computationally intensive language models on this task.


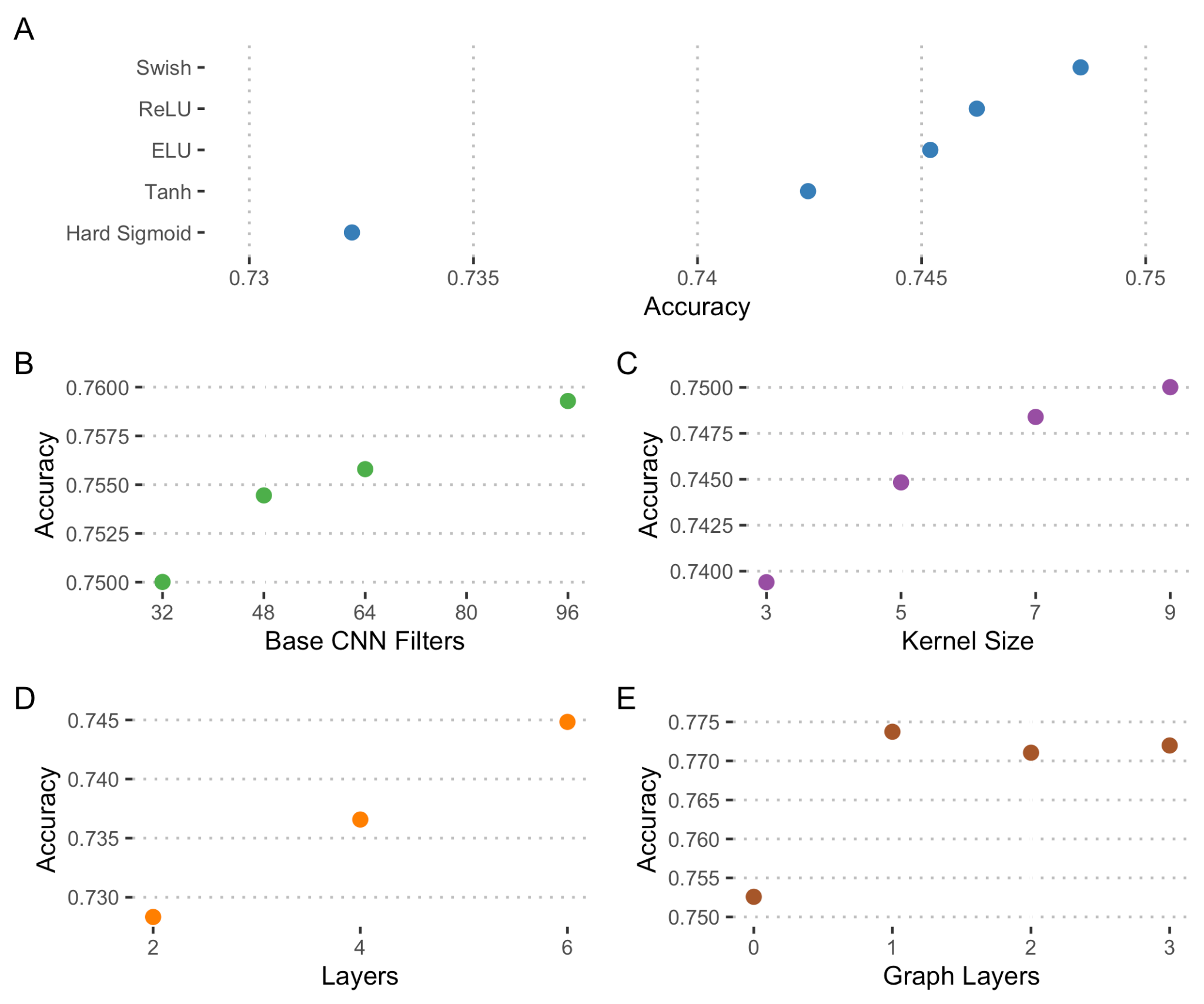


**Figure S1** - Hyperparameter optimisation. These results are from optimising the frequency classification model but equivalent results occurred when optimising the PSSM predictor. **A**: CNN activation function. **B**: Number of CNN filters. **C**: Size of CNN kernel. **D**: Number of UNET compression layers. **E**: Number of graph CNN layers.


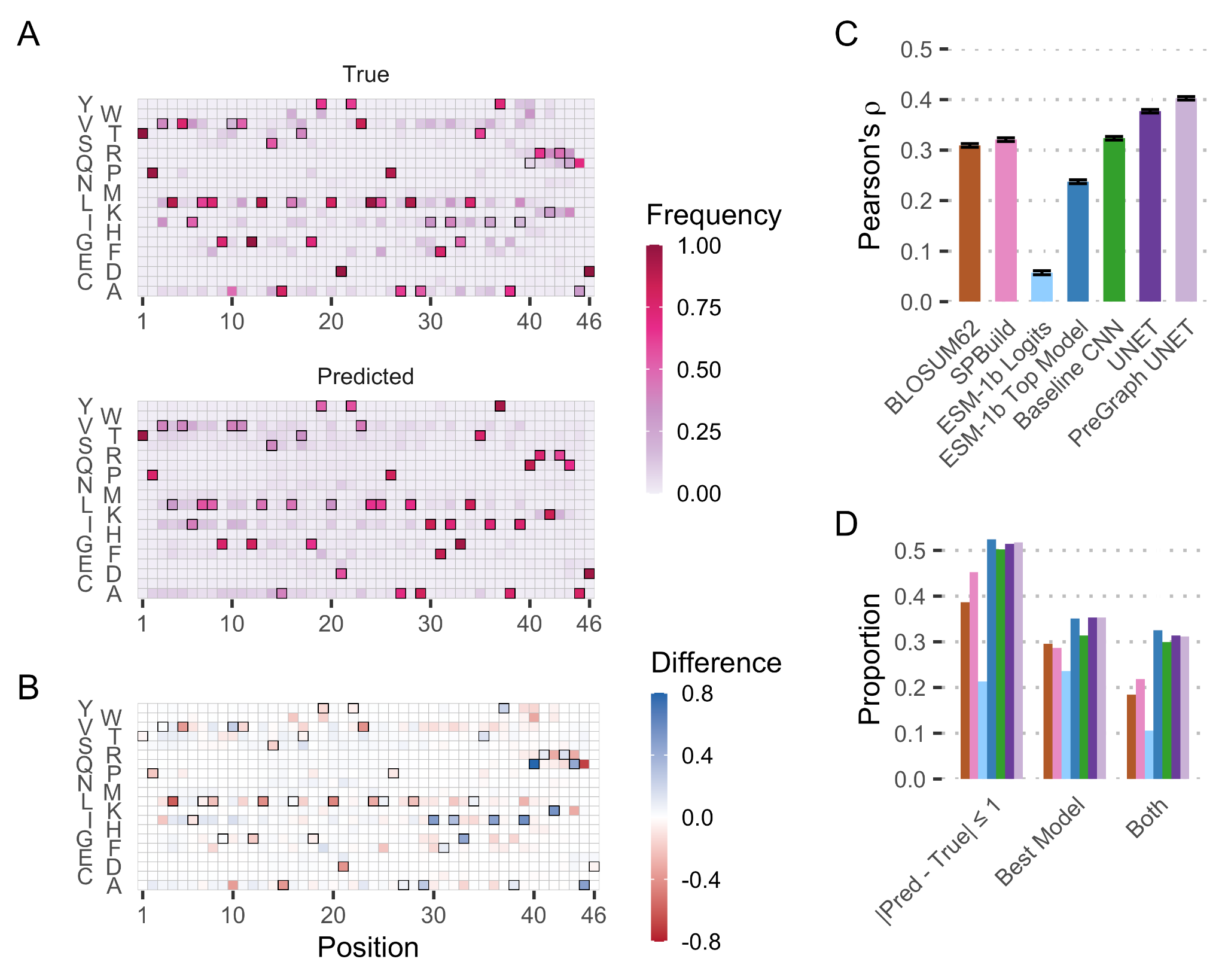


**Figure S2** - PSSM Prediction **A**: Example true and predicted MSA frequency predictions from the Sequence UNET model for the bacterial mercury transporter MerF. The wild-type amino acid at each position is outlined. **B**: Difference between predicted and true values of the PSSMs in **A**. **C**: Pearson correlation between predicted and true PSSM values for Sequence UNET, a single layer CNN, SPBuild, ESM-1b logits, an ESM-1b top model and BLOSUM62 based on the CASP12 test set. Confidence intervals are based on Fisher's Z transform of the correlation coefficient. **D**: PSSM prediction comparison showing proportion of predictions within 1 unit of the true value, that are the best or equal best and both. The models are coloured as in **C**.

# Variant frequency classification

The model can also be trained as a frequency classifier, using a binary cross-entropy loss function and a sigmoid activation function in the final layer. This outputs a score predicting whether each variant occurs below a specific frequency threshold (**Fig. S3A**), which can be treated as a soft deleteriousness score under the assumption that deleterious variants occur at low frequencies across homologous proteins. There is a significant separation between high and low frequency variants in test set proteins (**Fig. S3B**).

We compared classification performance with: a baseline single layer CNN; BLOSUM62 scores to represent an evolutionary baseline; SIFT4G [12] scores, which are based on an MSA and approximate deleteriousness; ESM-1b logits; and a single layer ESM-1b representation top model trained on CASP12 95% thinned ProteinNet data. We only compare to one VEP (SIFT4G) because while variant frequency is related to deleteriousness it is not exactly the same, meaning most VEPs are not directly comparable. We compare the generalised pathogenicity predictor model more widely. SIFT4G results were generated for the ProteinNet test set using default parameters and aligning against UniRef90. Sequence UNET and baseline CNN results were again generated using the python package. The single sigmoid layer ESM-1b top model was again trained on ProteinNet CASP12 95% thinned training data and scores predicted from the test set as well as ESM-1b logit results.

Sequence UNET matches the ESM-1b top model and outperforms all other models on the ProteinNet test set proteins when using models’ natural deleteriousness thresholds. Again, GraphCNN features slightly improve performance (**Fig. S3C**). It has higher average accuracy and precision than the other predictors as well as a higher median F1 score, indicating a better balance between precision and recall. This analysis highlights that SIFT4G is tuned for recall, having very high recall but low precision. Finally, Sequence UNET has a higher average Cohen's κ score, indicating performance that is further improved over random chance. In contrast, BLOSUM62, SIFT4G and ESM-1b logits can be little better than chance for many proteins.

Different frequency thresholds lead to very different training outcomes (**Fig. S3D**), with a difference in accuracy over 10% between tested thresholds. The difference in performance likely reflects the difficulty of determining the status of intermediate frequency variants, which are not obviously incompatible amino acids but also do not follow common motifs. Different thresholds can be potentially valuable for different applications, for example the 0.1 threshold could be useful for identifying variants very likely to be neutral and conversely low thresholds can identify very rare mutations that are likely to be deleterious. We primarily used a threshold of < 0.01 for further tests, reasoning that this is a good cut-off for general deleteriousness and that optimisations to this problem, which appears to be the most difficult, will carry over to other thresholds as well.

A ROC analysis gives a better overview of overall predictor characteristics, testing performance over a range of deleteriousness thresholds. The final model has a ROC AUC of 0.81 (**Fig. S3E**), making it much better at frequency classification than SIFT4G and BLOSUM62 scores (both AUC = 0.61). Adding structural features again improves performance a small amount (AUC = 0.83), resulting in a very similar performance profile to the ESM-1b top model (AUC = 0.83). A similar pattern is observed in precision recall analysis, with our model performing best (auPR = 0.79/0.81) alongside the ESM-1b top model (auPR = 0.8) (**Fig. S3F**). SIFT4G likely performs poorly on this task, compared with reasonable performance elsewhere, because the SIFT4G score is calculated by normalising modelled MSA frequency against the frequency of the most frequent amino acid at that position, which allows a convenient consistent deleteriousness cut-off across positions but throws away information about the absolute value of frequencies. Scores from EVE and DeepSequence would also likely suffer from this problem. Conversely ESM-1b logits seem likely to be good at determining likely amino acids at a position but are poor at determining those that are particularly rare, explaining poor PSSM and low frequency classification performance.


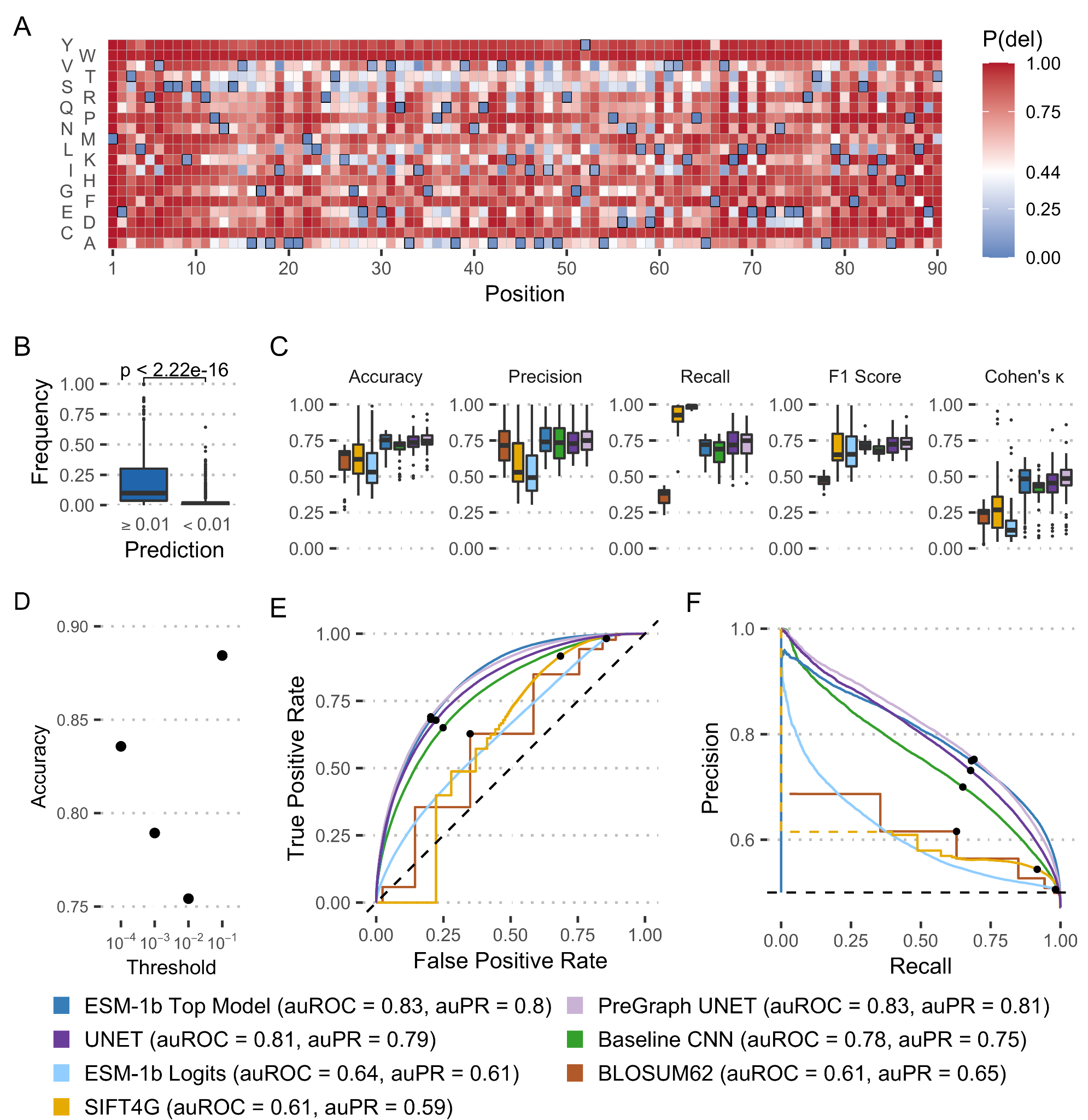


**Figure S3** - Variant Frequency Classification **A**: Example of sequence-based frequency classification (f < 0.01) on *T. thermophilus* protein SPOVS. The deleteriousness threshold (P(del) > 0.44) was chosen to balance specificity and sensitivity by maximising $\sqrt{(1-FPR)^{2}+TPR^{2}}$(see **E**). **B**: Observed MSA frequency distribution of variants in SPOVS predicted to be above and below 0.01 frequency. **C**: Performance statistics comparing Sequence UNET with and without structural features, ESM-1b logits, an ESM-1b top model, a baseline single layer CNN, SIFT4G and BLOSUM62. The first four are at their natural deleteriousness thresholds (0.5 for the neural networks & 0.05 for SIFT4G) and the optimal threshold for BLOSUM62 was determined to be -2 based on the ROC analysis. Each boxplot shows the distribution of performance statistics over the different proteins in the ProteinNet Casp12 95% thinned test dataset. **D**: Frequency classification accuracy of models trained at different deleterious frequency thresholds. **E/F**: ROC (**E**) and PR (**F**) curves comparing Frequency classification (< 0.01) performance of the models in **C**. The standard model deleteriousness thresholds are marked with black points on the curves.

# Model Generalisation Dataset Biases

The ClinVar dataset has a narrow focus, containing information on human variants with validated clinical significance, leading to biases in protein composition with less than 2000 well studied human proteins represented and 14 proteins each with over 100 variants together accounting for 15.4% of the dataset variants. Despite these biases a random split was sufficient to get variants from a broad range of proteins in each set, with proteins and positions exclusively in each set and no single protein dominating. This suggests the model cannot just learn the properties of individual positions or proteins, especially as it is only trained for a few epochs. However, the limited range and type of protein included in the dataset does suggest that the fine-tuning will not necessarily transfer to other families of proteins or those from other species. There are also proteins with variants included in training and test sets, which introduces a risk that the model learns specific features of the protein rather than general pathogenicity - effectively overfitting on protein rather than variant. However, the short training time, early stopping, limited number of trainable weights and mix of pathogenic and benign variants from each protein makes this unlikely to be a significant factor in results. In our experiments we do not observe training and validation losses diverging at the point of early stopping.

Deep mutational scanning experiments are performed on proteins from a range of species, although often expressed in a model system such as yeast. The correlation between predicted scores and DMS measurements varies a lot between studies (**Fig. S4**), likely due to variation in experimental techniques and the selection pressure applied. For example, artificial selection for a specific and sometimes unnatural trait will not necessarily lead to good correlations with natural fitness effects. Sequence UNET and its derivatives were found to perform similarly well across different species, suggesting the models can be usefully applied in a range of contexts and isn’t specific to human or mammalian proteins, even when fine-tuned on ClinVar.


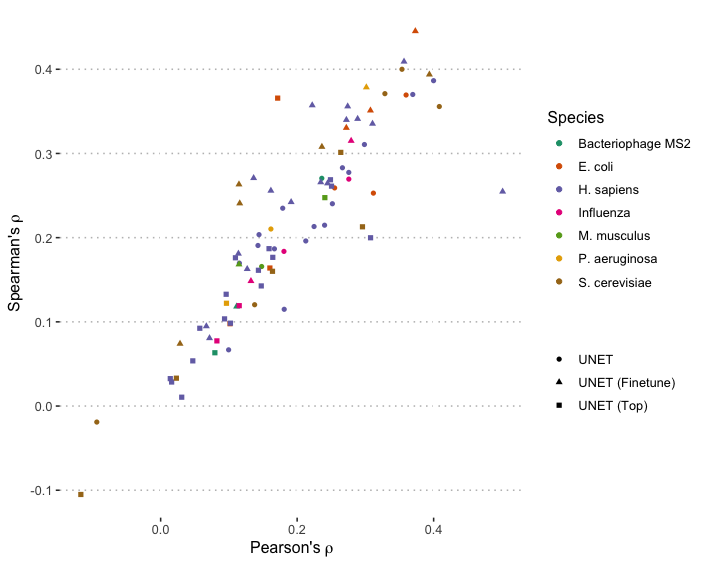
**Figure S4** - Pearson and Spearman’s Rank correlation between Sequence UNET predictions and DMS scores across different studies, coloured by species.

# Prediction Variation with Variable Padding

The model supports padding sequences with additional zeroes to allow proteins of different lengths to be processed in batches; a fact that is utilised to speed up training and can speed up predictions. This is in addition to the mandatory padding required to make most proteins lengths divisible by $2^{5}$. Additional padding does alter prediction calculation but was included during training so the network would be expected to learn to account for variable length. This is generally found to be the case, although in a small number of cases changing padding changes variant predictions significantly. We tested the impact of padding by making predictions for proteins in the ProteinNet CASP12 validation set with 32 to 1024 amino acids of additional padding, testing both the frequency classifier and PSSM predictor models. In general, the padded predictions correlated very strongly with unpadded predictions (**Fig. S5A**). Correlation decreased a reasonable amount in a few shorter proteins, particularly for the classifier model, but predictions in the vast majority of proteins correlated with $\rho> 0.9$. Correlation also decreases, albeit modestly in most cases, as the amount of additional padding increases, suggesting it is beneficial to group similar length variants in batches to minimise the required padding. It is also rare for predictions to change classification, with the altered prediction crossing the 0.5 boundary (**Fig. S5B**). Again, it is shorter proteins that tend to have the most variants changing class. Overall, the average difference between padded and unpadded predictions is generally modest, particularly for the PSSM predictor (**Fig. S5C**). The majority of predictions (> 70%) from the classifier still change by less than 0.05 with over 85% less than 0.1. Finally, additional padding is found to have a negligible effect on overall accuracy (**Fig. S5D**), including at positions near to the end of the protein which are generally affected more by padding changes. Overall, adding additional padding to make batch predictions does have a large impact on a small number of predictions but is unlikely to impact overall results, especially in the large-scale analysis this tool is most suited to. This allowed us to use batching for our large-scale analyses without impacting predictions too much. In future, adapting training to batch together similar length proteins and using a similar strategy during prediction would help to reduce this effect even further. It might also be beneficial to add a minimum padding to standardise results on shorter proteins, which are generally most affected.


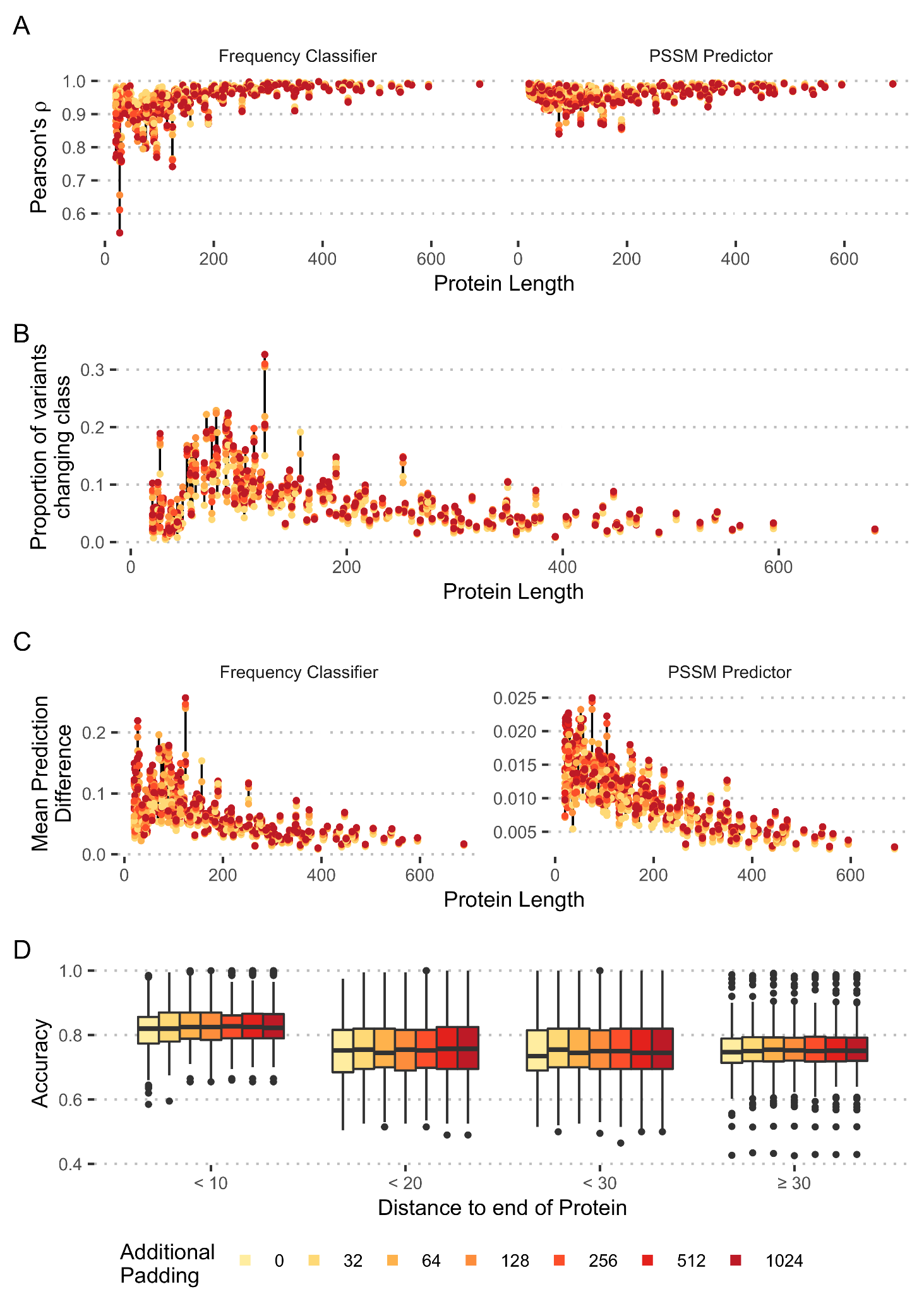


**Figure S5** - Impact of padding on predictions. **A**: Pearson correlation between unpadded predictions and those made on sequences with varying levels of additional padding. Correlations were calculated for each protein independently using both the PSSM predictor and frequency classifier model. **B**: Proportion of variants predicted to change class ($p\leq0.5$ to $p>0.5$ or vice-versa) when different levels of additional padding are added to sequences, using the frequency classifier model. **C**: Mean difference between unpadded and padded predictions on a range of proteins using both models. **D**: Accuracy of frequency classification in different proteins with various levels of additional padding, stratified by the distance between the position and the protein end, where padding begins. All panels are based on proteins from the ProteinNet CASP12 validation set. In **A**, **B** and **C** points from the same protein at different pad levels are linked by lines.

# References

1. Dunham A S, Beltrao P, AlQuraishi M. Sequence UNET v1.0.0. 2023; doi:10.5281/zenodo.7621269

2. Dunham AS, Beltrao P, AlQuraishi M. Sequence UNET. https://github.com/allydunham/sequence_unet. Accessed 14 April 2023

3. Dunham AS, Beltrao P, AlQuraishi M. Sequence UNET Weights. https://www.ebi.ac.uk/biostudies/studies/S-BSST732. Accessed 24 November 2021

4. Dunham AS. ProteinNetPy. https://github.com/allydunham/proteinnetpy. Accessed 14 April 2023

5. Dunham AS. ProteinNetPy v0.5.3. 2023; doi:10.5281/zenodo.7621273

6. Yamada KD, Kinoshita K. De novo profile generation based on sequence context specificity with the long short-term memory network. BMC Bioinformatics. 2018; doi:10.1186/s12859-018-2284-1

7. Biegert A, Söding J. Sequence context-specific profiles for homology searching. Proc Natl Acad Sci USA. 2009; doi:10.1073/pnas.0810767106

8. Angermüller C, Biegert A, Söding J. Discriminative modelling of context-specific amino acid substitution probabilities. Bioinformatics. 2012; doi:10.1093/bioinformatics/bts622

9. Boratyn GM, Schäffer AA, Agarwala R, Altschul SF, Lipman DJ, Madden TL. Domain enhanced lookup time accelerated BLAST. Biology Direct. 2012; doi:10.1186/1745-6150-7-12

10. Rives A, Meier J, Sercu T, Goyal S, Lin Z, Liu J, et al. Biological structure and function emerge from scaling unsupervised learning to 250 million protein sequences. Proceedings of the National Academy of Sciences. Proceedings of the National Academy of Sciences; 2021; doi:10.1073/pnas.2016239118

11. Meier J, Rao R, Verkuil R, Liu J, Sercu T, Rives A. Language models enable zero-shot prediction of the effects of mutations on protein function [preprint]. bioRxiv. 2021; doi:10.1101/2021.07.09.450648

12. Vaser R, Adusumalli S, Leng SN, Sikic M, Ng PC. SIFT missense predictions for genomes. Nature Protocols. 2015; doi:10.1038/nprot.2015.123
